# Selection of References for microRNA Quantification in Japanese Flounder (*Paralichthys olivaceus*) Normal Tissues and *Edwardsiella tarda-*Infected Livers

**DOI:** 10.3390/genes13020175

**Published:** 2022-01-19

**Authors:** Saisai Liu, Haofei Song, Zeyu Liu, Wei Lu, Quanqi Zhang, Jie Cheng

**Affiliations:** 1 Key Laboratory of Marine Genetics and Breeding, Ocean University of China, Ministry of Education, 5 Yushan Road, Qingdao 266003, China; liusaisai97@163.com (S.L.); m17860736515@163.com (H.S.); liuzeyu_ouc@163.com (Z.L.); lw1981@ouc.edu.cn (W.L.); qzhang@ouc.edu.cn (Q.Z.); 2 Laboratory for Marine Fisheries Science and Food Production Processes, Pilot National Laboratory for Marine Science and Technology (Qingdao), 1 Wenhai Road, Qingdao 266237, China; 3 Laboratory of Tropical Marine Germplasm Resources and Breeding Engineering, Sanya Oceanographic Institution, Ocean University of China, Sanya 572024, China

**Keywords:** microRNA, reference gene, qRT-PCR, *Paralichthys olivaceus*, *Edwardsiella tarda*

## Abstract

MicroRNA (miRNA) plays essential roles in post-transcriptional regulation of protein coding genes, and the quantitative real-time polymerase chain reaction (qRT-PCR) is the powerful and broadly employed tool to conduct studies of miRNA expression. Identifying appropriate references to normalize quantitative data is a prerequisite to ensure the qRT-PCR accuracy. Until now, there has been no report about miRNA reference for qRT-PCR in Japanese flounder (*Paralichthys olivaceus*), one important marine cultured fish along the coast of Northern Asia. In this study, combined with miRNA-Seq analysis and literature search, 10 candidates (*miR-34a-5p*, *miR-205-5p*, *miR-101a-3p*, *miR-22-3p*, *miR-23a-3p*, *miR-210-5p*, *miR-30c-5p*, *U6*, *5S rRNA*, and *18S rRNA*) were chosen as potential references to test their expression stability among *P. olivaceus* tissues, and in livers of *P. olivaceus* infected with *Edwardsiella tarda* at different time points. The expression stability of these candidates was analyzed by qRT-PCR and evaluated with Delta CT, BestKeeper, geNorm, as well as NormFinder methods, and RefFinder was employed to estimate the comprehensive ranking according to the four methods. As the result, *miR-22-3p* and *miR-23a-3p* were proved to be the suitable combination as reference miRNAs for both *P. olivaceus* normal tissues and livers infected with *E. tarda*, and they were successfully applied to normalize *miR-7a* and *miR-221-5p* expression in *P. olivaceus* livers in response to *E. tarda* infection. All these results provide valuable information for *P. olivaceus* miRNA quantitative expression analysis in the future.

## 1. Introduction

MicroRNA (miRNA) is the small non-coding RNA (ncRNA) with a length of about 22–24 nucleotide (nt), which exists broadly in diverse organisms and plays important regulatory roles in gene expression at the post-transcription level [1]. Mature miRNA pairs completely or incompletely with mRNA 3’ un-translated region (UTR) through the 2–7 nt seed region to degrade mRNA or inhibit translation [2]. Since miRNA was first found in *Caenorhabditis elegans*, an increasing number of studies have reported that miRNA could regulate various biological processes, such as cell proliferation and differentiation [3,4], embryonic development [5,6], immunology [7], reproduction [8], as well as stress responses [9], in many eukaryotes.

To explore the function of miRNA, its expression pattern commonly needs to be verified. There are several methods to quantify miRNA expression, including northern blot, microarray, and quantitative real-time PCR (qRT-PCR) [10,11,12]. qRT-PCR is one of the most widely used technique to quantify miRNA expression due to several advantages, such as good accuracy, high sensitivity, quick reaction, broad application, as well as low cost [13]. However, differences in RNA quality and reverse transcription efficiency may lead to inaccuracy in qRT-PCR. Therefore, it is essential to employ an appropriate reference to normalize the expression of the target RNA molecules [14].

Endogenous reference of the same RNA class can better normalize the expression level of targeting RNA [15]. Recently, small ncRNA families, including the small nuclear RNA (snRNA) or the small nucleolar RNA (snoRNA), as well as the ribosomal RNA (rRNA), are the most frequently used references for miRNA quantification in vertebrates [16], such as small nuclear RNA *U6* (~107 nt) and *5S rRNA* (~121 nt). However, it is vital that the references employed for normalization should generally have a similar length, structure, as well as the same nature of the interest targets to assure accurate results, including the comparable efficiency in RNA isolation, cDNA synthesis, and quantification in qRT-PCR [17]. Moreover, recently, there were studies reported that *U6* and *5S rRNA* displayed less stable than some miRNAs across different tissues and experimental conditions, indicating the inadvisable to use *U6* or *5S rRNA* as the reference without evaluation [18].

However, at present, the application of evaluated miRNA as qRT-PCR reference has not been commonly adopted, although it is essential that the references selected need to have the similar nature as the studied molecules. In this manner, limited studies have verified the expression stability of certain miRNAs in model organisms [13,15,18,19], as well as in teleost species. For example, in common carp (*Cyprinus carpio.* var), when considering different tissues and developmental stages, *let-7a* and *miR-23a* were proved to be the most stable miRNAs [16]; in Atlantic salmon (*Salmo salar*), *miR-25-3p* and *miR-455-5p* were the most suitable combination as references among tissues and with infections [20]; *miR-101a* was verified to be the stable miRNA among different tissues and developmental stages of grass carp (*Ctenopharyngodon idella*) [21]; *miR-22* and *miR-23a* were the most suited candidates for various tissues and developmental stages in Chinese perch (*Siniperca chuatsi*) [22]. Moreover, in Atlantic cod (*Gadus morhua*), *miR-17-1-5p*, *miR-25-3p*, and *miR-210-5p* were the best combinations for different tissues [23], and in blunt snout bream (*Megalobrama amblycephala*), *miR-221-3p* and *miR-103-3p* were the best combination for different tissues and various stresses [24].

Japanese flounder (*Paralichthys olivaceus*) is one of the most widely cultured marine fish along the coast of Northern China as well as in Japan and Korea. Until now, most studies about the expression of Japanese flounder protein-coding genes with qRT-PCR usually use *β-actin* and *ubiquitin-conjugating enzyme* (*UBCE*) as references [25,26], whereas studies about miRNA expression commonly use *U6* as reference [27], with no report about the evaluation of miRNA reference. Therefore, the goal of this study was to systematically choose and evaluate suitable references for normalizing miRNA qRT-PCR among Japanese flounder normal tissues as well as in livers with bacterial infection. We first selected 10 candidate references according to miRNA-Seq data and a literature survey, and then identified the most suitable reference miRNAs across adult tissues and bacterial infectious conditions with qRT-PCR. Our findings suggest using appropriate reference miRNAs, which will be beneficial in qRT-PCR studies of miRNA expression in Japanese flounder under normal and infectious conditions.

## 2. Materials and Methods

### 2.1. Sample Collection

Nine tissue samples (heart, liver, spleen, kidney, brain, gill, intestine, ovary and testis) were collected from three 2-years old normal Japanese flounder, with ovaries and testes collected from six 2-years old Japanese flounder. Bacterial challenge experiments were described previously by Liu et al. [27]. Briefly, Japanese flounder individuals were collected and acclimated at 19 °C aerated seawater (pH 7.5, salinity 30 ppt) for one week, and were classified into three experimental groups, as the blank control (BC) group, Ringer’s solution (RS) group, and *Edwardsiella tarda*-challenged (EC) group. The BC group was not treated with any solution, while RS and EC groups were intraperitoneally injected with 1 mL Ringer’s solution or *E. tarda* suspension (2 × 10^7^ CFU/mL), respectively. After 3 and 24 h post injection, the livers from EC and RS groups were sampled. Three individuals from each time point of the BC, RS and EC groups were sampled. A final 15 individuals were collected, and about 3 g of liver was sampled and stored at −80 °C for RNA isolation later.

### 2.2. Candidate miRNA References Selection

The candidate miRNA references for Japanese flounder were selected through several ways.

#### 2.2.1. miRNA-Seq for Normal Tissues and Bacterial Infected Livers of Japanese Flounder

We previously generated miRNA-Seq data for 11 adult tissues (brain, kidney, gill, heart, liver, spleen, stomach, muscle, intestine, testis, and ovary) of Japanese flounder, as well as miRNA-Seq data for 15 liver tissues (triplicate for BC-0h, RS-3h, RS-24h, EC-3h, and EC-24h) [27]. For each tissue sample, only miRNAs with the expression level higher than five transcripts per million (TPM) were included for the expression stability analyses due to the difficulty to detect and quantify low-expressing miRNAs with qRT-PCR. Two methods were employed to analyze the miRNA expression stability: the fold change (FC) comparison [28] and the coefficient of variation (CV) evaluation [29].

For the FC method, log_2_FC was calculated in pairwise samples to compare the differential expression [30]. MiRNAs with a high log_2_FC (≥1 among 11 tissue types and ≥1.2 in differentially treated livers) were excluded from the list of stably expressed miRNAs (Supplementary Table S1). For the CV method, the mean expression (mean), standard deviation (SD), as well as the CV of each miRNA were calculated. Mean and SD were evaluated among the 11 normal tissues or differentially treated livers with three biological replicates, respectively. CV was then represented as SD/mean.

#### 2.2.2. Published miRNA References for Teleost Species

A few conserved miRNAs have been verified as qRT-PCR references in the studies of teleost species (Table S2). For example, *miR-101a* for different tissues and developmental stages in grass carp (*C. idella*) [21]; *miR-22* and *miR-23a* for different tissues and developmental stages in Chinese perch (*S. chuatsi*) [22]; *miR-210-5p* for different tissues in Atlantic cod (*G. morhua*) [23]; *miR-23a-5p* for different tissues and various stress in Atlantic salmon (*S. salar*) [31]; and *miR-23a* for different tissues and developmental stages in common carp (*C. carpio.* var) [16]. Some miRNAs from the studies above (Supplementary Table S2) were differentially expressed among Japanese flounder tissues, including *let-7a*, *miR-25-3p*, *miR-99-5p*, and *miR-17*, and they were excluded from further analysis.

#### 2.2.3. Commonly Used miRNA References for qRT-PCR: *U6*, *5S rRNA*, and *18S rRNA*

Furthermore, *5S rRNA*, *18S rRNA*, as well as *U6* were also included to be evaluated as references due to their common usage in miRNA qRT-PCR. In summary, *miR-34a-5p*, *miR-205-5p*, *miR-101a-3p*, *miR-22-3p*, *miR-23a-3p*, *miR-210-5p*, *miR30c-5p*, *U6*, *5S rRNA*, and *18S rRNA* were finally chosen to evaluate their expression stability in the following analysis.

### 2.3. RNA Extraction and miRNA cDNA Synthesis

Total RNA was first isolated with TRIzol^TM^ reagents (Invitrogen, Carlsbad, CA, USA). After RNA isolation and purification, NanoPhotometer Pearl (Implen, Munich, Germany) and 1.5% agarose gel electrophoresis were employed to measure the concentration and integrity of the RNA. MiRNA cDNA was synthesized by the Mir-X^TM^ miRNA First-Strand Synthesis Kit (clontech, Mountain View, CA, USA) with the reaction volume of 10 μL. After the reaction, 90 μL DEPC treated H_2_O was added to make the final concentration of miRNA cDNA 10 ng/μL, and then stored in −20 °C.

### 2.4. qRT-PCR Analysis

The expression of the selected candidate references was quantified by qRT-PCR. The forward primers of *miR-34a-5p*, *miR-205-5p*, *miR-101a-3p*, *miR-22-3p*, *miR-23a-3p*, *miR-210-5p*, and *miR30c-5p* were the same as their mature miRNA sequences (replace U with T, Table 1). The reverse miRNA primer was the mRQ 3′ primer from the Mir-X^TM^ miRNA First-Strand Synthesis Kit (clontech, Mountain View, CA, USA). Primers for *5S rRNA*, *18S rRNA*, and *U6* were retrieved from studies related to miRNA expression [24,32,33]. qRT-PCR was carried out in 384-well plates with 2 × SYBR Green qPCR Master Mix (US Everbright^®^ Inc., Suzhou, Jiangsu, China) using the LightCycler^®^ 480 real time PCR system (Roche Molecular Biochemical, Mannheim, Germany). Each sample was conducted with three biological replicates and each biological replicate with three technical repetitions. Standard curves were established from 10-fold serial dilutions with the pool of miRNA cDNA of all samples. The total volume of each reaction system was 20 μL, including 2 μL miRNA cDNA (5 ng/μL), 10 μL LightCycler^®^ 480 SYBR Green I Master, 0.4 μL of each primer (10 μM), and 7.2 μL DEPC treated H_2_O. The reaction program was 95 °C 5 min, followed by 40 cycles of 95 °C for 15 s and 60 °C for 20 s.

### 2.5. Statistical Analysis

The melting curves and cycle threshold (Ct) values (Supplementary Table S3) of qRT-PCR were exported from the LightCycler^®^ 480 real time PCR system software, and the performance of each candidate reference was evaluated according to the Delta CT [34], BestKeeper [35], NormFinder [36], and geNorm [37] algorithm. RefFinder (https://www.heartcure.com.au/reffinder/, accessed on 18 March 2021) was also employed to rank the comprehensive stability of the candidate references [38].

### 2.6. Reference miRNA Validation

To confirm the reliability of the potential reference genes, *miR-7a* and *miR-221-5p* were selected from our previous miRNA-Seq study [27], because they exhibited upregulated expression after *E. tarda* infection in Japanese flounder liver. The expression of miRNAs was normalized with both the most and the least stable references in *E. tarda*-infected Japanese flounder livers. The conditions of qRT-PCR were the same as those described above. The relative expression of both miRNAs was computed based on the 2^−ΔCt^ method.

## 3. Results

### 3.1. Candidate Reference Selection for Japanese Flounder miRNA Quantification

**Figure 1:**
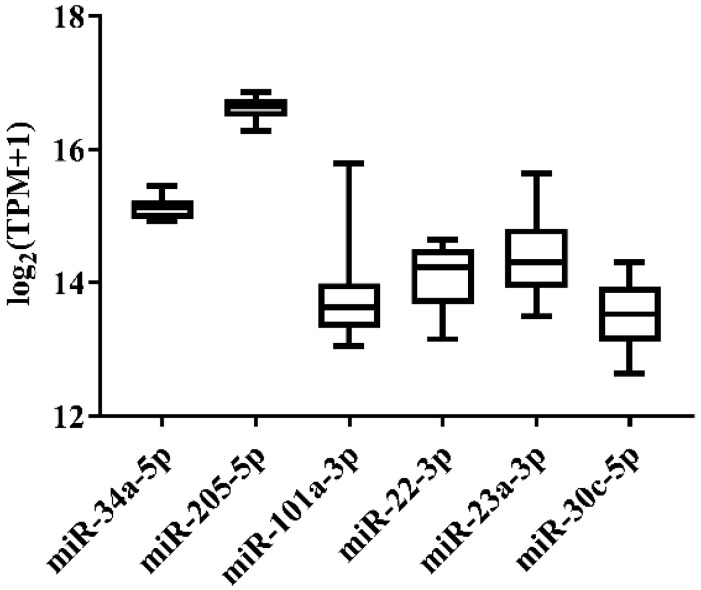
Expression variation of miRNA reference candidates among 11 normal tissues of Japanese flounder from miRNA-Seq data.

**Figure 2:**
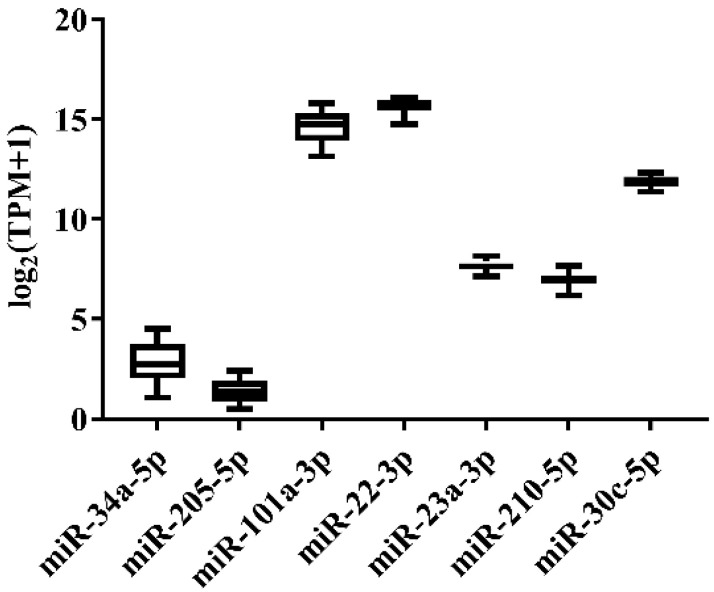
Expression variation of miRNA reference candidates in the livers of Japanese flounder injected with *E. tarda* or Ringer’s solution from miRNA-Seq data.

In order to select the candidate qRT-PCR reference miRNA, two methods (log_2_FC and CV) were first performed to evaluate miRNA expression variation across miRNA-Seq data of 11 Japanese flounder normal tissues and livers infected with Ringer’s solution or *E. tarda*. Two criteria were employed: (a) exclusion of differentially expressed miRNAs through the log_2_FC pairwise comparison of tissues or experimental groups (Supplementary Table S1); (b) inclusion of miRNA with the lowest overall CV across all tissues or experimental groups (Supplementary Table S1). For Japanese flounder normal tissues, subject to log_2_FC < 1, the CV values were ranked, and *miR-34a-5p* (CV = 0.1230) and *miR-205-5p* (CV = 0.1278) with the lowest CV were selected as the candidate references (Figure 1 and Supplementary Table S1). For Japanese flounder livers infected with *E. tarda* or Ringer’s solution at 3 h and 24 h, subject to log_2_FC < 1.2, *miR-202-3p* (CV = 0.1093), *miR-30c-5p* (CV = 0.1659), *miR-203a-5p* (CV = 0.1702), and *miR-23a-3p* (CV = 0.1817) were the most stably expressed among the experimental groups (Figure 2 and Supplementary Table S1). However, the expression levels of *miR-202-3p* and *miR-203a-5p* among the infection experimental groups were lower than one TPM (Supplementary Table S1), and thus, they were not suitable as references and were excluded from the further analysis. Therefore, *miR-30c-5p* and *miR-23a-3p* were included as the candidates.

According to the survey of published miRNA references in teleost species (Supplementary Table S2), such as in Atlantic salmon (*S. salar*) [20], grass carp (*C. idella*) [21], Chinese perch (*S. chuatsi*) [22], common carp (*C. carpio.* var) [16], and Atlantic cod (*G. morhua*) [23], *miR-101a-3p*, *miR-22-3p*, *miR-23a-3p*, *miR-210-5p*, and *18S rRNA* represented relatively high expression stability among Japanese flounder tissues, and they showed conserved sequence identity among teleost species as well. *5S rRNA* and *U6* were also included, as they were commonly used as references in studies about miRNA expression [27,33]. In summary, a total of 10 reference candidates were selected, including *miR-34a-5p*, *miR-205-5p*, *miR-101a-3p*, *miR-22-3p*, *miR-23a-3p*, *miR-210-5p*, *miR-30c-5p*, *U6*, *5S rRNA*, and *18S rRNA*, in Japanese flounder for the following qRT-PCR analysis. The primer sequences of these candidate references are shown in Table 1.

The reverse primer employed for miRNA quantification was universal primer (mRQ 3′ Primer, Takara).

### 3.2. Amplification Efficiency of the Candidate References by qRT-PCR

Amplification efficiency of the 10 selected candidates was analyzed by the standard curve method, with 10-fold serial dilutions of a pool for all samples (10^1^ ng/μL, 10^0^ ng/μL, 10^−1^ ng/μL, 10^−2^ ng/μL, and 10^−3^ ng/μL) as templates to perform qRT-PCR, respectively. As a result, the amplification efficiency of the candidate references in Japanese flounder was from 83% to 204% (Table 1), and *miR-205-5p*, *miR-101a-3p*, *miR-22-3p*, and *miR-23a-3p* generally complied with the normal requirements of about 90–110%.

### 3.3. Expression Stability of the Candidate References among Normal Tissues of Japanese Flounder by qRT-PCR

**Figure 3:**
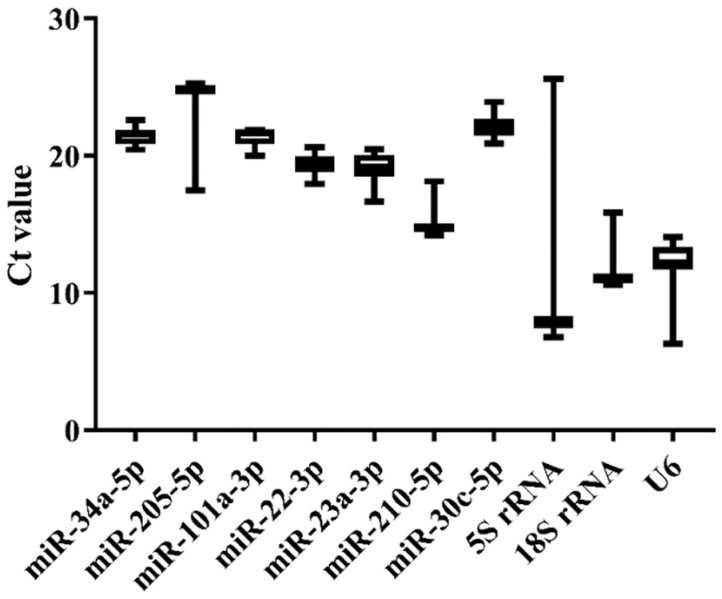
Ct value variability for the candidate references in normal tissues of Japanese flounder by qRT-PCR.

We selected nine tissues (heart, liver, spleen, brain, kidney, gill, intestine, testis, and ovary) of normal Japanese flounder to estimate the expression stability of the 10 candidate references by qRT-PCR. The melting curves of the candidates from qRT-PCR indicated that *miR-34a-5p*, *miR-205-5p*, *miR-22-3p*, *miR-23a-3p*, and *5S rRNA* generally represented a single peak with specific product among tissues (Supplementary Figure S1). The Ct values from qRT-PCR also varied among the 10 candidate references (Figure 3 and Supplementary Table S3). The commonly used genes such as *5S rRNA*, *18S rRNA*, and *U6* showed higher expression level (low Ct value) and lower stability (high Ct value variation) than other miRNA candidates, among which *5S rRNA* displayed the highest variation of Ct values (Figure 3). Moreover, *miR-34a-5p*, *miR-101a-3p*, *miR-22-3p*, *miR-23a-3p*, and *miR-30c-5p* presented lower variation of Ct values, indicating their higher expression stability (Figure 3). Among these candidates with high stability in Ct values, *miR-22-3p* and *miR-23a-3p* had lower Ct values, indicating their higher expression level.

Furthermore, Delta CT, BestKeeper, NormFinder, and geNorm were employed to calculate and sort the expression stability of candidate references (Table 2). As a result, *miR-22-3p*, *miR-23a-3p*, and *miR-101a-3p* were the most stable miRNAs by Delta CT, NormFinder, and geNorm methods, while *miR-34a-5p* was the best from BestKeeper. *5S rRNA* and *U6* were the least stable candidates among Japanese flounder tissues with the lowest ranking values. For the geNorm stability value (M), all candidate references were less than 1.5 except *5S rRNA* and *U6* (Table 3), as the most stable reference should have the lowest M value. According to the comprehensive ranking of RefFinder and M value of geNorm, *miR-22-3p* and *miR-23a-3p* were recommended as the most suitable combined references for miRNA quantification among normal Japanese flounder tissues (Table 2 and Table 3).

### 3.4. Expression Stability of Candidate References in Japanese Flounder Livers Injected with E. tarda or Ringer’s Solution by qRT-PCR

**Figure 4:**
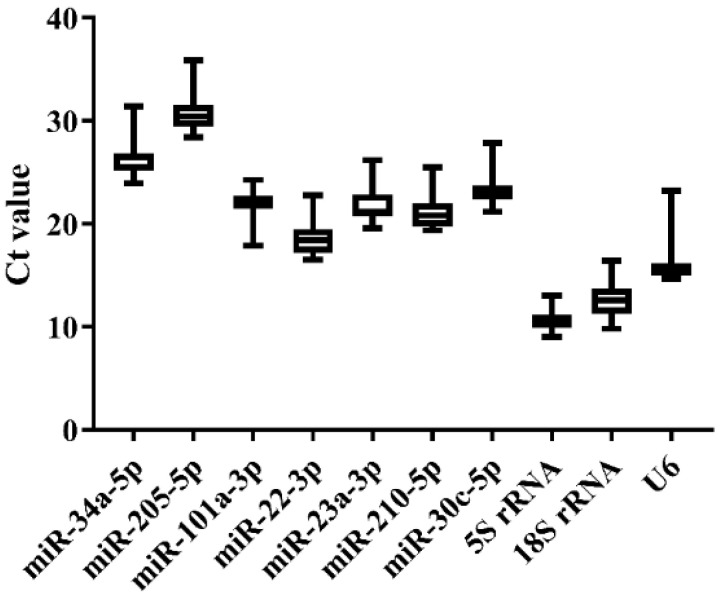
Ct value variability for the candidate references in Japanese flounder livers with Ringer’s solution or *E. tarda* injection at different time points by qRT-PCR.

Three groups of liver samples in the challenge experiment, including the blank control group (BC-0h), Ringer’s solution group (RS-3h and -24h), and *E. tarda*-challenged group (EC-3h and -24h), were used for qRT-PCR analysis. As a result, melting curves displayed that *miR-101a-3p*, *miR-22-3p*, *miR-23a-3p*, *5S rRNA*, and *18S rRNA* generally presented a single peak (Supplementary Figure S2). The Ct value variation among experimental groups was generally similar across the 10 candidate references (Figure 4 and Supplementary Table S3). Consistent with normal tissues, the expression level of *5S rRNA*, *18S rRNA*, and *U6* was higher (low Ct values) than other miRNA references, while *miR-34a-5p* and *miR-205-5p* were lowly expressed (high Ct values) across all groups (Figure 4). The stability of the 10 candidates was calculated and ranked by the Delta CT, BestKeeper, NormFinder, and geNorm methods, and the comprehensive ranking was carried out by the RefFinder method. As a result, *miR-22-3p*, *miR-23a-3p*, and *miR-210-5p* were the steadiest miRNAs by the Delta CT, NormFinder, and geNorm methods, while *5S rRNA* was the most stable one by BestKeeper (Table 4). The M value of *5S rRNA* was greater than 1.5, which was not appropriate as a reference (Table 5). According to the stability order and melting curves, *miR-22-3p* and *miR-23a-3p* were still the best reference combination in Japanese flounder livers injected with *E. tarda* and Ringer’s solution (Table 4 and Table 5).

### 3.5. Reference miRNA Validation in Japanese Flounder Livers Infected with E. tarda by qRT-PCR

**Figure 5:**
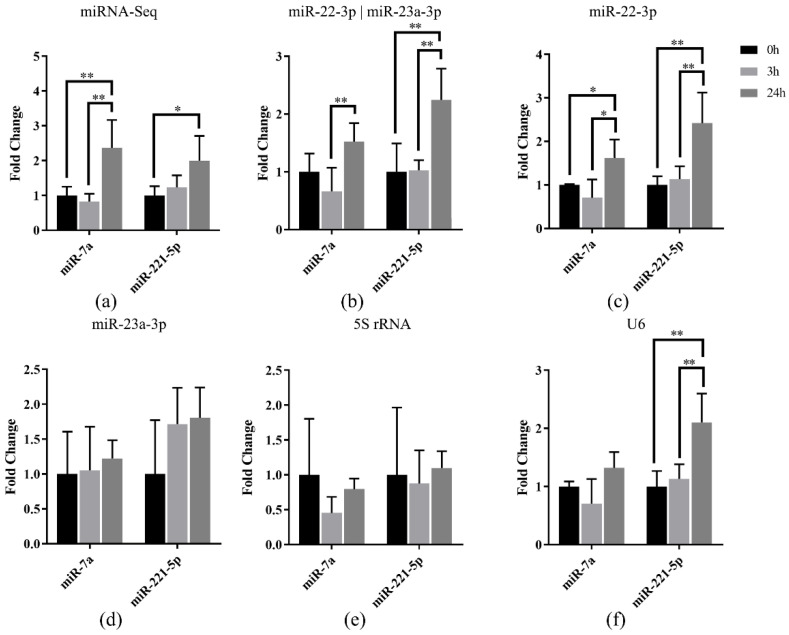
Relative quantification of *miR-7a* and *miR-221-5p* expression in livers of Japanese flounder infected with *E. tarda* at different time points by (**a**) miRNA-Seq and qRT-PCR referenced with (**b**) *miR-22-3p* and *miR-23a-3p*, (**c**) *miR-22-3p*, (**d**) *miR-23a-3p*, (**e**) *5S rRNA*, and (**f**) *U6*, respectively. Data are shown as mean ± SD (*n* = 3). Asterisks indicate statistical significance between groups (* *p* < 0.1 and ** *p* < 0.05).

To validate the selected miRNA references in Japanese flounder, the relative expression of *miR-7a* and *miR-221-5p* were normalized using the selected references in Japanese flounder livers with *E. tarda* infection. Our previous miRNA-Seq result [27] showed that *miR-7a* and *miR-221-5p* were upregulated in livers after 3 h or 24 h infection of *E. tarda* (Figure 5a). Data normalization using the most stably expressed references, *miR-22-3p* and the combination of *miR-22-3p* and *miR-23a-3p*, resulted in generally consistent miRNA expression patterns along the infection time points (Figure 5b,c), whereas the result referenced with only miR-23a-3p was not as stable as the combined references (Figure 5d).

Moreover, the results normalized using the unstable reference genes *5S rRNA* and *U6* showed that there was no induced expression of *miR-7a* and *miR-221-5p* after infection referenced with *5S rRNA* (Figure 5e), while there was induced expression only for *miR-221-5p* when *U6* was the reference (Figure 5f). The above results indicated that the most stable reference (*miR-22-3p*) and the most suitable combination (*miR-22-3p* and *miR-23a-3p*) showed similar miRNA expression patterns with miRNA-Seq data, so that they could be used for normalization of miRNA in Japanese flounder.

## 4. Discussion

MiRNA plays important regulatory roles in almost all aspects of development and physiology. It is inevitable to invest the miRNA expression pattern across different samples if we need to study its function. Although there are several approaches, qRT-PCR is one of the most widely applied techniques to evaluate miRNA expression. The accuracy of the qRT-PCR result could be easily affected due to the sensitivity of the experimental process; therefore, a suitable reference is required to normalize qRT-PCR data to ensure the validity of miRNA expression level [13,14]. Commonly used in miRNA qRT-PCR, the references are relatively short genes, such as *5S rRNA*, *18S rRNA*, and *U6* [39,40]. There has been a lot of controversies about the stability of these commonly used references, and many experiments have reported that their expression was not constant across different species, tissues, and experimental treatments, which made them unsuitable as default miRNA references [23,24,26,31]. Hence, the expression level and stability were examined for 10 potential references in Japanese flounder normal tissues as well as livers under experimental conditions to reduce avoidable errors in miRNA qRT-PCR analysis. Owing to discrepant statistical algorithms, the ranking of candidate references obtained by the three methods (Delta CT, NormFinder, and geNorm) was generally similar, but different from the result from BestKeeper. Consistent with the argument above, *U6*, *5S rRNA*, and *18S rRNA* all performed less stability with way much higher expression levels than *miR-22-3p* and *miR-23a-3p* in both normal tissues and infected livers, only with *5S rRNA* performing better with the BestKeeper method (Table 2 and Table 4). In addition, the amplification efficiency of the three commonly used reference genes (*U6*, *5S rRNA*, and *18S rRNA*) were all lower than 90%, while the ideal value should be around 90–110%. Another reason that *rRNA* and *U6* are unsuitable as a reference is that their expression levels are much higher than most miRNAs, which could lead to the deviated qRT-PCR result of targeting miRNA with low expression [22,41]. In our analysis, *U6* and *rRNA* genes represented the highest expression among all candidate references (Figure 3 and Figure 4). To sum up, *U6*, *5S rRNA*, and *18S rRNA* are not suitable as references for miRNA quantification in Japanese flounder.

In general, no single reference gene can be stable across all samples [42], which emphasizes the significance of identifying adequate references to normalize miRNA expression under various experiment conditions. For example, in common carp (*C. carpio*), *5S rRNA* and *18S rRNA* were deemed as the most suitable references for normalizing miRNA expression during early developmental stages, whereas *let-7a* and *miR-23a* were the best combination in different developmental gonads [16]. In Chinese perch (*S. chuatsi*), there were various suitable references under different conditions. For instance, among different tissues, the best combination was *miR-22-3p* and *miR-23a-3p*, and for developmental stages of muscle, the most appropriate references were *let-7a* and *miR-26a*, whereas for different embryonic developmental stages, the suited combination was *miR-22-3p* and *miR-146a*, and for fasting-refeeding treated livers, the best combination was *miR-26a* and *miR-23a* [22]. In Japanese flounder, the expression stability of candidate references in normal tissues and *E. tarda*-infected livers were analyzed, and the three best performers in normal tissues were identified as *miR-22-3p*, *miR-23a-3p*, and *miR-101a-3p*, whereas in *E. tarda*-infected livers, the best candidates were *miR-22-3p*, *miR-23a-3p*, and *miR-210-5p*. However, *miR-101a* and *miR-210-5p* were discarded due to their non-specific amplification and high amplification efficiency (Table 1 and Supplementary Figure S1). Therefore, *miR-22-3p* and *miR-23a* were concluded to be the best combination of reference miRNAs for Japanese flounder in both normal tissues and *E. tarda*-infected livers, which were also successfully validated in Japanese flounder livers for *miR-7a* and *miR-221-5p* expression in response to *E. tarda* infection (Figure 5).

## 5. Conclusions

In this study, 10 candidate references were identified for Japanese flounder miRNA quantification through both miRNA-Seq data and literature search. With qRT-PCR and statistical evaluation, commonly used *U6* and *5S rRNA* were not suitable as references, whereas *miR-22-3p* and *miR-23a-3p* were the most stably expressed miRNAs in both Japanese flounder normal tissues and livers challenged by *E. tarda*, which could be the most suitable combined references for Japanese flounder miRNA quantification in future studies.

## Figures and Tables

**Table 1:** Primer sequences and amplification efficiency of the candidate references.

Genes	Primer Sequence (5′–3′)	Amplification Efficiency
*miR-34a-5p*-F	TGGCAGTGTCTTAGCTGGTTGT	173.84%
*miR-205-5p*-F	TCCTTCATTCCACCGGAGTCTG	90.74%
*miR-101a-3p*-F	TACAGTACTGTGATAACTGAAG	96.22%
*miR-22-3p*-F	AAGCTGCCAGCTGAAGAACTGT	99.98%
*miR-23a-3p*-F	ATCACATTGCCAGGGATTTCCA	110.00%
*miR-210-5p*-F	AGCCACTGACTAACGCACATTG	131.01%
*miR-30c-5p*-F	TGTAAACATCCTTGACTGGAAGCT	220%
*5S rRNA*-F	CCATACCACCCTGAACAC	83.38%
*5S rRNA*-R	CGGTCTCCCATCCAAGTA	
*18S rRNA*-F	CCTGAGAAACGGCTACCACAT	85.45%
*18S rRNA*-R	CCAATTACAGGGCCTCGAAAG	
*U6*-F	TTGGAACGATACAGAGAAGATTAGC	86.42%

**Table 2:** Expression stability ranking of the candidate references for normal tissues of Japanese flounder. The best performers, *miR-22-3p* and *miR-23a-3p*, are shown in bold.

Method	Ranking Order (Better–Good–Average)									
Delta CT	***miR-22-3p***	***miR-23a-3p***	*miR-101a-3p*	*miR-30c-5p*	*miR-34a-5p*	*miR-210-5p*	*18S rRNA*	*miR-205-5p*	*U6*	*5S rRNA*
BestKeeper	*miR-34a-5p*	*miR-101a-3p*	***miR-22-3p***	*miR-30c-5p*	*miR-210-5p*	***miR-23a-3p***	*18S rRNA*	*U6*	*miR-205-5p*	*5S rRNA*
NormFinder	***miR-22-3p***	*miR-210-5p*	***miR-23a-3p***	*miR-30c-5p*	*miR-101a-3p*	*18S rRNA*	*miR-34a-5p*	*miR-205-5p*	*U6*	*5S rRNA*
geNorm	***miR-22-3p|miR-23a-3p***		*miR-101a-3p*	*miR-34a-5p*	*miR-30c-5p*	*miR-210-5p*	*18S rRNA*	*miR-205-5p*	*U6*	*5S rRNA*
Recommended comprehensive ranking	***miR-22-3p***	***miR-23a-3p***	*miR-101a-3p*	*miR-34a-5p*	*miR-30c-5p*	*miR-210-5p*	*18S rRNA*	*miR-205-5p*	*U6*	*5S rRNA*

**Table 3:** Expression stability values (M) calculated by geNorm for normal tissues of Japanese flounder.

geNorm	*miR-22-3p|miR-23a-3p*	*miR-101a-3p*	*miR-34a-5p*	*miR-30c-5p*	*miR-210-5p*	*18S rRNA*	*miR-205-5p*	*U6*	*5S rRNA*
geNorm Stability value (M)	0.652	0.770	0.899	0.997	1.123	1.263	1.493	1.736	2.514

**Table 4:** Expression stability ranking of the candidate references in Japanese flounder livers with Ringer’s solution or *E. tarda* injection at different time points. The best performers, *miR-22-3p* and *miR-23a-3p*, are shown in bold.

Method	Ranking Order (Better--Good--Average)									
Delta CT	*miR-210-5p*	***miR-22-3p***	***miR-23a-3p***	*miR-205-5p*	*miR-30c-5p*	*18S rRNA*	*miR-34a-5p*	*U6*	*miR-101a-3p*	*5S rRNA*
BestKeeper	*5S rRNA*	*miR-101a-3p*	*miR-34a-5p*	*miR-30c-5p*	*miR-210-5p*	*18S rRNA*	*miR-205-5p*	***miR-22-3p***	***miR-23a-3p***	*U6*
NormFinder	*miR-210-5p*	***miR-22-3p***	***miR-23a-3p***	*miR-205-5p*	*miR-30c-5p*	*18S rRNA*	*miR-34a-5p*	*U6*	*miR-101a-3p*	*5S rRNA*
geNorm	***miR-22-3p|miR-23a-3p***		*miR-210-5p*	*18S rRNA*	*miR-205-5p*	*miR-30c-5p*	*U6*	*miR-34a-5p*	*miR-101a-3p*	*5S rRNA*
Recommended comprehensive ranking	*miR-210-5p*	***miR-22-3p***	***miR-23a-3p***	*miR-205-5p*	*miR-30c-5p*	*18S rRNA*	*miR-34a-5p*	*5S rRNA*	*miR-101a-3p*	*U6*

**Table 5:** Expression stability values (M) calculated by geNorm in Japanese flounder livers with Ringer’s solution or *E. tarda* injection at different time points.

geNorm	*miR-22-3p|miR-23a-3p*	*miR-210-5p*	*18S rRNA*	*miR-205-5p*	*miR-30c-5p*	*U6*	*miR-34a-5p*	*miR-101a-3p*	*5S rRNA*
geNorm Stability value (M)	0.292	0.356	0.622	0.811	0.942	1.102	1.284	1.396	1.510

## Data Availability

The raw sequencing data was obtained at NCBI with accession number SRP135934. The TPM values and expression results of miRNAs were in Supplementary Table S1.

## Supplementary Materials

The following are available online at https://www.mdpi.com/article/10.3390/genes13020175/s1, Table S1. MiRNA reference selection from miRNA-Seq data with CV and log_2_FC methods. Table S2: Literature survey of candidate miRNA for qRT-PCR in other teleost species. Table S3: Ct values generated from miRNA qRT-PCR of Japanese flounder normal tissues and livers injected with *E. tarda* and Ringer’s solution. Figure S1: Melting curves of the candidate references in normal tissues of Japanese flounder by qRT-PCR. Figure S2: Melting curves of the candidate references in Japanese flounder livers with Ringer’s solution and *E. tarda* injection at different times by qRT-PCR.
