# Supplementary material for: Selection of References for microRNA Quantification in Japanese Flounder (Paralichthys olivaceus) Normal Tissues and Edwardsiella tarda-Infected Livers
Source: Genes (Basel). 2022 Jan 19;13(2):175. doi: 10.3390/genes13020175 (PMC8871525; doi:10.3390/genes13020175)
Supplement: Supplementary file 1 [file genes-13-00175-s001.zip › Table S2-Candidate miRNAs in teleost.pdf]

Table S2. Reference survey of candidate miRNA for qRT-PCR in other teleost species.

species	Best reference	application condition	citation
Atlantic Cod (*Gadus morhua*)	miR-25-3p, miR-210-5p	9 tissues under normal condition	Andreassen, 2016
Atlantic salmon (*Salmo salar*)	miR-25-3p, miR-455-5p	7 tissues under normal condition<br>individuals with infectious salmon anemia	Johansen and Andreassen, 2014
Blunt snout bream (*Megalobrama amblycephala*)	miR-221-3p, miR-103-3p	9 tissues under normal condition<br>heat stress, ammonia stress, bacterial challenge, glycolipid stress	Liu, 2018
Yellow River Carp (*Cyprinus carpio. var*)	5S	11 tissues of adult and juvenile individual	Wang, 2019
	5S, 18S	6 early different developmental stages (morula -- primordial gonad)	
	Let-7a, miR-23a	3 developmental stages (primordial gonad -- adult carp gonad)	
grass carp (*Ctenopharyngodon idella*)	miR-101a	12 tissues under normal condition	Xu, 2014
	miR-126-3p, miR-101a, miR-192, miR-451, miR-22a	bacterial infection (Aeromonas hydrophila S2)	
	miR-126-3p, miR-22a	18 developmental stages (unfertilized eggs -- 10 days post-hatching)	
Atlantic salmon (*Salmo salar*)	miR-99-5p, miR-23a-5p	hypoxic stress	Zavala, 2017
Chinese Perch (*Siniperca chuatsi*)	miR-22a, miR-23a	8 tissues under normal condition	Zhu, 2015
	miR-22a, miR-146a	8 embryonic developmental stages (two-cell stage -- larval stage)	
	let-7a, miR-26a	6 post-embryonic developmental stages of muscle (20 -- 150 dph)	
	miR-26a, miR-23a	starvation	
