# Supplementary figures and images for: Selection of References for microRNA Quantification in Japanese Flounder (Paralichthys olivaceus) Normal Tissues and Edwardsiella tarda-Infected Livers
Source: Genes (Basel). 2022 Jan 19;13(2):175. doi: 10.3390/genes13020175 (PMC8871525; doi:10.3390/genes13020175)

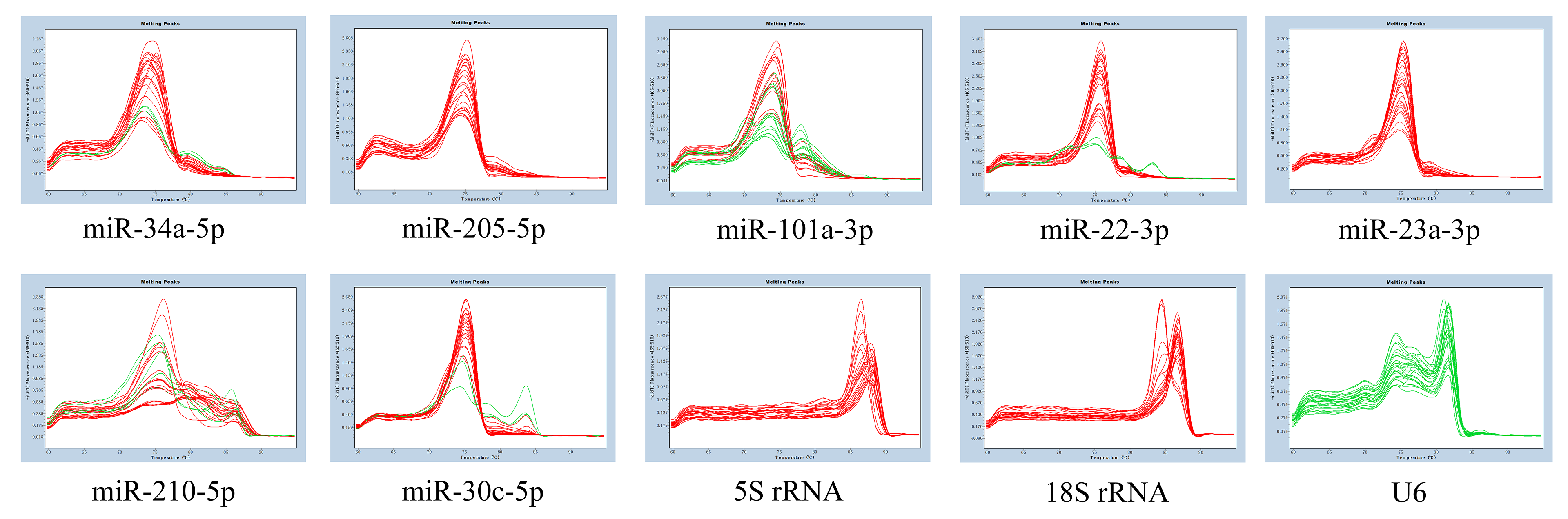

Supplement: Supplementary file 1 [file genes-13-00175-s001.zip › FigS1.qRT-PCR-melting curves-tissues2.tif]

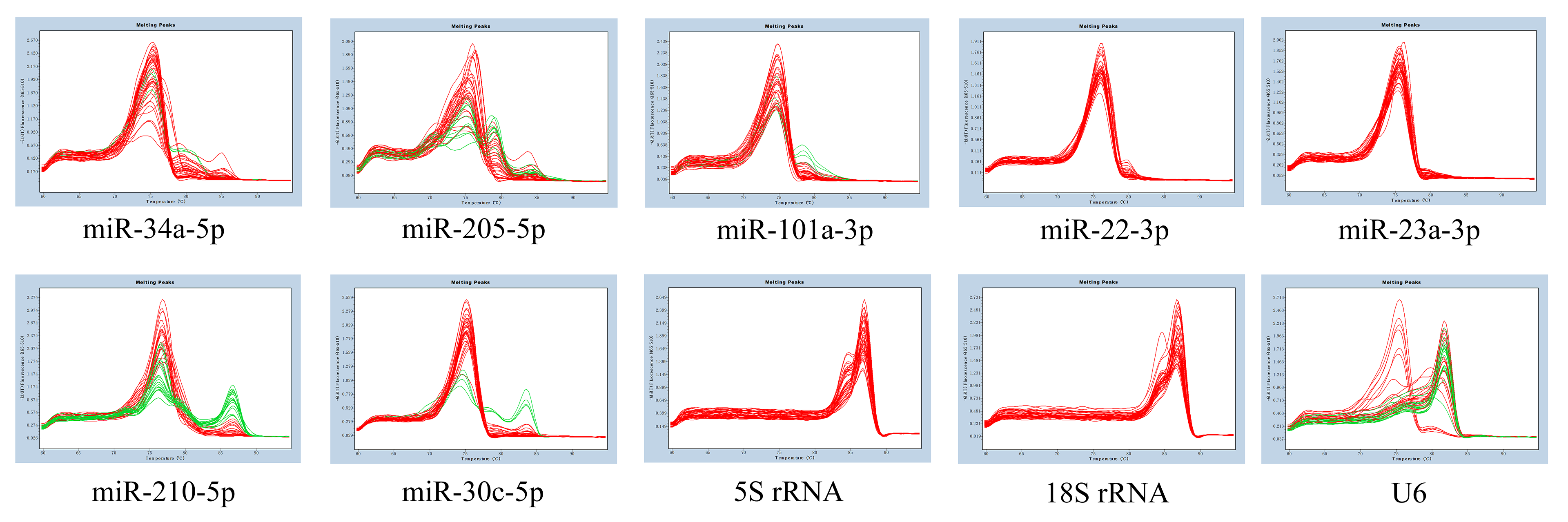

Supplement: Supplementary file 1 [file genes-13-00175-s001.zip › FigS2.qRT-PCR-melting curves-E.tarda2.tif]
